# FGFR Testing in Metastatic Urothelial Carcinoma—Who, When, and How to Test

**DOI:** 10.3390/cancers18030444

**Published:** 2026-01-29

**Authors:** André Mansinho, José Carlos Machado, Cátia Faustino, Arnaldo Figueiredo, João Moreira Pinto, Nuno Vau, João Ramalho-Carvalho, Manuel R. Teixeira

**Affiliations:** 1 START Lisbon, Hospital de Santa Maria, Unidade Local de Saúde Santa Maria, 1649-035 Lisbon, Portugal; andre.mansinho@startlisbon.com; 2 GIMM–Gulbenkian Institute for Molecular Medicine, 1649-028 Lisbon, Portugal; 3 i3S—Instituto de Investigação e Inovação em Saúde, University of Porto, 4200-135 Porto, Portugal; josem@ipatimup.pt; 4 Institute of Molecular Pathology and Immunology, University of Porto (IPATIMUP), 4200-135 Porto, Portugal; 5 Medical Faculty, University of Porto, 4200-319 Porto, Portugal; 6 Department of Medical Oncology, Instituto Português de Oncologia do Porto, 4200-072 Porto, Portugal; catia.faustino@ipoporto.min-saude.pt; 7 Faculdade de Medicina, Universidade de Coimbra, 3004-528 Coimbra, Portugal; ajcfigueiredo@ulscoimbra.min-saude.pt; 8 Urology and Transplantation Department, Centro Hospitalar e Universitário de Coimbra, 3004-561 Coimbra, Portugal; 9 Department of Medical Oncology, Hospital da Luz, 1500-650 Lisboa, Portugal; joao.pmoreira.pinto@hospitaldaluz.pt; 10 Department of Medical Oncology, Hospital Beatriz Ângelo, 2674-514 Loures, Portugal; 11 Urologic Oncology, Champalimaud Clinical Center, 1400-038 Lisbon, Portugal; nuno.vau@fundacaochampalimaud.pt; 12 Johnson & Johnson Innovative Medicine, Lagoas Park, 2740-262 Porto Salvo, Portugal; jramalh3@its.jnj.com; 13 Department of Laboratory Genetics, Portuguese Oncology Institute of Porto (IPO Porto), Porto Comprehensive Cancer Center, 4200-072 Porto, Portugal; 14 Cancer Genetics Group, IPO Porto Research Center (CI-IPOP)/RISE@CI-IPOP (Health Research Network), Portuguese Oncology Institute of Porto (IPO Porto)/Porto Comprehensive Cancer Center, 4200-072 Porto, Portugal; 15 Department of Pathology and Molecular Immunology, School of Medicine and Biomedical Sciences (ICBAS), University of Porto, 4050-313 Porto, Portugal

**Keywords:** biomarker, *FGFR* alteration, metastatic urothelial carcinoma, molecular testing, precision oncology, targeted therapy

## Abstract

Metastatic urothelial carcinoma (mUC) is a lethal cancer with limited therapeutic options. Advances in genomic and transcriptomic research have deepened the understanding of mUC biology, leading to the identification of clinically relevant molecular alterations that represent potential actionable targets. This has broadened the treatment landscape of the disease to include novel agents, such as antibody–drug conjugates (e.g., enfortumab vedotin) and targeted therapies, including the pan-fibroblast growth factor receptor (FGFR) inhibitor erdafitinib. Genomic alterations in *FGFR3* are well-established oncogenic drivers in bladder cancer and represent predictive biomarkers of response to FGFR-targeted therapies. The phase III THOR trial demonstrated the clinical benefit of erdafitinib in previously treated mUC patients harboring *FGFR3* alterations and supported its subsequent approval by the European Medicines Agency. In this context, accurate molecular profiling is essential to guide patient selection for FGFR inhibitor therapy. Equally important is the standardization and timely implementation of *FGFR3* testing in clinical practice to optimize treatment planning. This review addresses key considerations in *FGFR3* testing in mUC and discusses how it can be routinely incorporated into clinical practice.

## Simple Summary

Bladder cancer that has spread to other parts of the body-called metastatic urothelial carcinoma (mUC)-is an aggressive disease with few effective treatments. Recent genetic research has revealed important changes in tumor DNA and gene activity that can represent targets for use of more precise treatments. One of these targets is a gene called *FGFR3*, which, when altered, can drive cancer growth. New drugs that specifically block this pathway, such as erdafitinib, have shown real benefits for patients whose tumors carry these *FGFR3* changes, as confirmed in a large international study (the THOR trial). Because of this, testing for *FGFR3* alterations has become an important step in deciding which patients might benefit from these treatments. This article aims to explain why and how *FGFR3* testing should be performed in everyday clinical practice, helping doctors select the right therapy for each patient. The findings could guide cancer centers on how to implement these tests more consistently and effectively, improving outcomes and advancing precision medicine in bladder cancer care.

## 1. Introduction

According to the latest GLOBOCAN data, bladder cancer is the ninth most common cancer worldwide, with an estimated 614,000 new cases and 220,000 deaths in 2022 and a considerably higher burden in men than women [1]. Urothelial carcinoma (UC) is the most common histologic subtype of bladder cancer, accounting for the vast majority of cases [2]. Although the 5-year relative survival rate for localized disease is 71.7%, it drops significantly to 8.3% for metastatic urothelial carcinoma (mUC) [3].

An estimated 5–10% of patients have metastatic disease at diagnosis [4]. A significant number of patients experience disease recurrence after radical cystectomy, with local relapse documented in approximately 30% of cases and distant metastases in up to 50% [5].

Over the past five years, the treatment landscape for mUC has evolved significantly with the introduction into the therapeutic armamentarium of immune checkpoint inhibitors (ICIs), antibody–drug conjugates (ADCs), and targeted therapies, both upfront and in subsequent lines. In fact, the European Society for Medical Oncology (ESMO) clinical practice guidelines currently recommend enfortumab vedotin–pembrolizumab as the preferred first-line therapy for advanced or metastatic UC irrespective of platinum eligibility, and nivolumab plus gemcitabine-cisplatin or platinum-based chemotherapy (gemcitabine plus cisplatin or carboplatin) for patients who are ineligible for enfortumab vedotin–pembrolizumab [6].

As the disease progresses, targeted therapies directed at fibroblast growth factor receptor (*FGFR*) alterations become an option for patients with tumors harboring some of these molecular aberrations [6], making testing for actionable genomic alterations in FGFR critical in the management of mUC patients. Accurate FGFR testing can guide personalized treatment strategies, optimize the use of FGFR inhibitors, and improve patient outcomes overall.

This article aims to provide an overview of the importance of *FGFR3* testing in mUC and offer recommendations on how to optimize and integrate it into routine clinical practice to guide personalized treatment decisions and improve patient care. The literature cited throughout the manuscript was selected based on the most recent and relevant publications available for each of the topics discussed.

## 2. Actionable Landscape in mUC

The introduction of ICIs, ADCs, and FGFR signaling pathway inhibitors has broadened the therapeutic landscape for mUC, opening the door for innovative and more effective treatment options [7,8]. While the advent of targeted therapies represents a major advance in the management of these malignancies, it also underscores the growing importance of biomarker testing to guide individualized treatment decisions.

In parallel, the field of biomarker research is rapidly evolving, with the goal of optimizing therapeutic selection based on the specific molecular characteristics of each patient’s tumor. Despite encouraging developments, the clinical utility of predictive biomarkers in clinical practice remains limited. Currently, only a few biomarkers—such as PD-L1 expression [9], *FGFR* gene alterations [7] and, more recently, HER2 status [10]—have gained regulatory or guideline-supported relevance for routine clinical decision making.

## 3. Overview of FGFR Alterations

The FGFR family consists of four transmembrane tyrosine kinase receptors—*FGFR1*, *FGFR2*, *FGFR3*, and *FGFR4*—that are integral to a wide range of biological processes, including embryonic development, regulation of angiogenesis, tissue homeostasis, and regulation of cell proliferation, survival, and migration [11].

**Figure 1:**
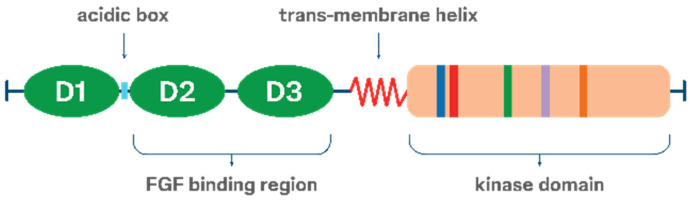
Structure of FGFRs.

FGFRs share a common structural architecture with other receptor tyrosine kinases. From the N- to the C-terminus, FGFRs consist of an extracellular domain, a single-pass transmembrane region, and an intracellular tyrosine kinase domain (Figure 1) [12,13]. The extracellular ligand-binding domain is composed of three immunoglobulin-like subdomains (D1, D2, and D3). Between D1 and D2 lies a highly conserved, aspartate-rich sequence known as the acid box, which plays a regulatory role in ligand binding and receptor autoinhibition [14].

**Figure 2:**
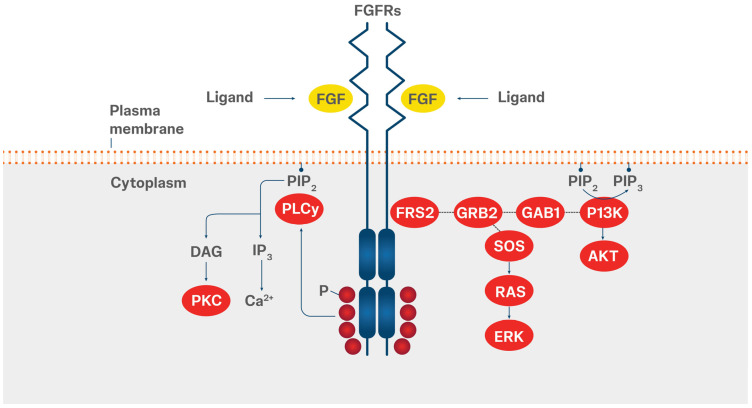
Mechanism of FGFR activation.

FGFR signaling is dysregulated in cancer patients due to various mechanisms, including gene fusions and rearrangements, point mutations, and amplifications in chromosomal or extrachromosomal DNA (Figure 2). Additionally, abnormal autocrine or paracrine secretion of fibroblast growth factor (FGF) ligands by stromal or cancer cells contributes to this aberrant activation [15,16]. *FGFR* fusions are classified into three types: type I and types II a and b (Table 1). Type I (non-receptor type) involves N-terminal replacement by fusion partners (*FGFR* is the 3′ fusion partner, the extracellular and the transmembrane domains are excluded from the fusion protein, which contains only the FGFR kinase domain linked to the 5′ protein partner), such as *BCR-FGFR1* and *ZMYM2-FGFR1*. Type II *FGFR* fusions involve receptor-type rearrangements in which the extracellular, transmembrane, and kinase domains of FGFR are maintained [17,18]. Type IIa fusions result from N-terminal replacement by fusion partners (e.g., *FN1-FGFR1* and *KLK2-FGFR2*), whereas type IIb fusions are characterized by C-terminal replacement by fusion partners (e.g., *FGFR2-BICC1* and *FGFR3-TACC3*) [17,18].

**Table 1:** Spectrum of *FGFR* alterations.

Amplification and/or Overexpression	Gain-of-Function Fusions			Gain-of-Function Point Mutations
	Type I	Type IIa	Type IIb	
Whole-gene amplifications	*BCR-FGFR1*	*FN1-FGFR3*	*FGFR1-TACC1*	FGFR1: N546K and K656E
Partial gene amplifications (excluding FGFR2 exon 18)	*CEP43-FGFR1*	*EPHA6-FGFR2*	*FGFR2-BICC1*	FGFR2: S252W, C382R, N549K and K656E
Enhancer amplifications or rearrangements	*CNTRL-FGFR1*	*FN1-FGFR2*	*FGFR2-CCDC6*	FGFR3: S249C, Y373C R248C, G370C, and K650E
	*MYO18A-FGFR1*	*KLK2-FGFR2*	*FGFR2-ERC1*	FGFR4: N535K, V550M and K645E
	*TPR-FGFR1*	*FN1-FGFR1*	*FGFR2-INA*	
	*TRIM24-FGFR1*		*FGFR2-SHTN1*	
	*ZMYM2-FGFR1*		*FGFR2-TACC2*	
	*ETV6-FGFR3*		*FGFR3-TACC3*	
	*FN1-FGFR3*			

## 4. Brief Overview of FGF/FGFR Signaling

FGFs are characterized by a conserved core domain with either a globular or atypical β-trefoil architecture and can be divided into three main categories: canonical FGFs (FGF1-10, FGF16-18, FGF20, and FGF22), endocrine FGFs (FGF19/FGF15, FGF21, and FGF23), and FGF-homologous factors (FGF11-14) [19,20,21]. The corresponding full-length FGF receptors (FGFR1-4) are type I transmembrane proteins characterized by three extracellular immunoglobulin-like domains and a cytoplasmic tyrosine kinase domain [22].

Collectively, FGF-FGFR signaling is critical for various cellular processes, including survival, proliferation, metabolism, migration, and differentiation, and also plays a key role in fetal development and maintenance of tissue and overall homeostasis. However, these signaling pathways can also contribute to tumor progression [18].

Activation of FGFRs occurs either through ligand-dependent dimerization or through pathological alterations that induce constitutive kinase activity by releasing autoinhibitory restraints, as illustrated in Figure 2 [23].

Alterations in FGFRs associated with cancer can drive tumorigenesis through dysfunctional signaling: *FGFR* amplifications lead to increased receptor expression and hyperactivation of downstream pathways; *FGFR* fusions or rearrangements can lead to fusion partner-induced dimerization, altered phosphorylation, and aberrant signaling cascades; mutations in the extracellular domain or transmembrane regions can affect FGFR activation by affecting ligand specificity or promoting ligand-independent dimerization; and alterations in the tyrosine kinase domain can result in constitutive FGFR activation by disrupting the inherent autoinhibitory mechanisms [23,24,25,26].

Next-generation sequencing (NGS) technology has been used to screen patients for potential actionable changes and identify alterations in cancer-related genes. *FGFR* gene alterations have been found in approximately 7.0% of unselected cancer patients [27,28]. *FGFR* alterations found in solid tumors include *FGFR1* amplifications in 20% of non-small cell lung cancers, *FGFR1/2* amplifications in 7–23% of breast cancers, *FGFR3* mutations in 10–60% and *FGFR3* fusions in 6% of UCs, *FGFR2* fusions in 10–20% of intrahepatic cholangiocarcinomas, *FGFR2* mutations in 12% of uterine endometrial cancers, and *FGFR2* amplifications in 5–10% of gastric cancers [16,18]. These variations illustrate the range of FGFR abnormalities found in cancer [16,18].

## 5. FGFR Alterations in Bladder Cancer

*FGFR* alterations are highly prevalent in UC. *FGFR3* has the highest frequency of alterations, followed by *FGFR1* and *FGFR2*, with an apparently similar age and gender distribution across the three subgroups [29,30]. *FGFR3* activating mutations and fusions and *FGFR3* overexpression have been found in bladder cancer [31]. Specifically, *FGFR3* mutations and fusions have been found in up to 80% of patients with non-muscle invasive bladder cancer (NMIBC) and approximately 10–20% of patients with muscle invasive bladder cancer (MIBC) [31]. Furthermore, FGFR3 overexpression has been reported in approximately 40–50% of MIBC patients [32], highlighting *FGFR3* as a promising target for bladder cancer therapy. In the THOR trial, the *FGFR3*-*S249C* mutation was the most common *FGFR* alteration (∼46%), followed by the *FGFR3*-*Y373C* mutation (17–19%) and the *FGFR3*-*TACC3_V1* fusion (10–12%) [7].

*FGFR3* alterations may be associated with the development of mUC [29,33,34]. *FGFR3* activation in UC occurs through mutations, overexpression or, less commonly, gene fusions [29,35,36]. Approximately 75–85% of tumors harboring *FGFR3* mutations show high levels of FGFR3 protein expression compared to only 22% of *FGFR3* wild-type tumors, and over 40% of tumors lacking detectable *FGFR3* mutations (*FGFR3* alteration-negative tumors) also exhibit FGFR3 overexpression [32,37]. In a recent study, *FGFR3* overexpression was shown to be exclusively associated with lower pathological stage and tumor grade [37]. Furthermore, among patients with *FGFR3* wild-type tumors, FGFR3 overexpression was not associated with disease-specific survival. In contrast, the presence of FGFR3 mutations identified patients with more favorable bladder cancer characteristics at cystectomy. Collectively, these findings support the concept that *FGFR3* mutations act as true oncogenic drivers and are biologically and functionally distinct from FGFR3 overexpression. Consequently, patients harboring *FGFR3* mutations are more likely to derive clinical benefit from FGFR-targeted therapies.

Although *FGFR3* alterations are widely recognized as clinically actionable events in mUC, their reported prevalence in the metastatic setting varies substantially across published series, typically ranging from approximately 10–20%, and occasionally beyond these limits [7,29,38]. This variability reflects not only biological heterogeneity but also important methodological and clinical factors that influence detection rates. On the one hand, series relying on archival primary tumor samples may overestimate or underestimate the prevalence of actionable *FGFR3* alterations in advanced disease because as tumors progress from localized to metastatic stages, clonal selection and tumor evolution may alter the detectable prevalence of *FGFR3*-driven clones [34,37,39] and metastatic lesions may therefore differ molecularly from the primary tumor, either due to selective outgrowth of *FGFR3*-wild-type subclones or treatment-induced evolutionary pressure [38,40,41]. On the other hand, prevalence estimates derived from heavily pretreated populations may differ from those obtained in treatment-naïve or earlier-stage cohorts, as prior treatments can reshape tumor clonal architecture [8,18,42], potentially leading to loss of *FGFR3*-altered clones, expansion of resistant *FGFR3*-wild-type populations, or reduced variant allele frequency below assay detection thresholds [16,41,43]. Differences in testing platforms and assay design also represent a potential source of variability due to different sensitivity for detection of some alterations [17,44,45]. The type and quality of analyzed specimens also affect detection rates. Metastatic biopsies often contain lower tumor cellularity than primary resections, and bone or small core biopsies may yield insufficient or degraded nucleic acids [46,47,48]. Lastly, many prevalence estimates originate from clinical trial populations or referral-based molecular profiling programs, which may not reflect unselected real-world mUC populations.

*FGFR3* point mutations are present in approximately 60% of NMIBC cases and typically correlate with lower tumor grade and stage [49]. The most common mutation, *S249C*, is thought to arise from apolipoprotein B mRNA editing catalytic polypeptide-like (APOBEC) protein activity [50]. *RAS* and *FGFR3* mutations are mutually exclusive and can be found in 90% of stage Ta tumors [51]. While activating point mutations in *FGFR3* are less common in MIBC compared to NMIBC [49], increased FGFR3 expression is common in MIBC [39]. Activating translocations involving *FGFR3* are observed in 2–5% of MIBC tumors [39]. In addition, FGFR1 expression may be upregulated or switched, potentially affecting epithelial–mesenchymal transition [52]. In metastatic tumors, *FGFR3* alterations are predicted to occur in approximately 15% of cases [38].

Genetic alterations in *FGFR3* have been associated with aberrant cellular transformation through activation of downstream signaling cascades, such as the MAPK and PI3K/AKT pathways, leading to cellular proliferation [29,53].

The role of these alterations in predicting cancer recurrence or progression remains unclear [53]. The identification of *FGFR3* alterations as potent oncogenic drivers in UC has led to the development of tyrosine kinase inhibitor (TKI) molecules targeting the FGFR transduction pathway. These TKIs can be either multi-targeting, non-selective, or selective, targeting multiple pathways or specifically the kinase domain of FGFRs, respectively. Erdafitinib is currently the only selective TKI approved for the treatment of patients with mUC harboring *FGFR3* genetic alterations (specifically, fusions *FGFR2-BICC1*, *FGFR2-CASP7*, *FGFR3-TACC3*, and *FGFR3-BAIAP2L* and point mutations R248C, S249C, G370C, and Y373C) [54]. The phase 3 THOR trial showed that erdafitinib therapy resulted in significantly longer overall survival than chemotherapy among patients with mUC and *FGFR* alterations after previous anti-PD-1 or anti-PD-L1 treatment [7]. Other FGFR inhibitors, including infigratinib [55] and rogaratinib, are being investigated in mUC [55]. The availability of these agents underscores the importance of accurately identifying *FGFR3* alterations to match patients to these therapies.

### Limitations of the Methodological and Testing Approaches Used in the FGFR Literature

The growing body of evidence supporting *FGFR* alterations as actionable targets in mUC has significantly advanced precision oncology. However, the methodologies underpinning this evidence present important limitations. A key issue is the heterogeneity of molecular testing approaches across studies. *FGFR* alterations have been detected using diverse platforms such as reverse transcription polymerase chain reaction (RT-PCR), DNA- and RNA-based NGS, fluorescence in situ hybridization (FISH), and occasionally immunohistochemistry (IHC), each with varying sensitivity for gene fusions and low-frequency variants. Earlier studies often relied on single-gene or hotspot assays, likely underestimating prevalence, whereas recent trials increasingly use comprehensive NGS panels. While these enable broader detection, they introduce variability in panel design, sequencing depth, bioinformatics, and reporting thresholds, complicating cross-study comparisons and limiting the generalizability of prevalence and outcome data.

Another critical factor is the type and timing of tumor samples. Most studies used archival primary tissue obtained months or years before FGFR-targeted therapy. Given the spatial and temporal heterogeneity of UC, this may not reflect the molecular profile of advanced disease. Clonal evolution under treatment pressure further raises uncertainty about the relevance of baseline FGFR status. Liquid biopsy offers a potential solution, but its application for FGFR detection remains inconsistent and lacks standardization.

## 6. Who and When to Test for FGFR Alterations

Genomic alterations in *FGFR3* are actionable biomarkers that inform targeted treatment strategies, ensuring that eligible patients receive optimal treatment. Currently, *FGFR3* alterations are the only validated biomarker routinely used in clinical practice for mUC [55], making testing for these alterations a cornerstone of personalized treatment in this setting.

The timing of FGFR testing is an important consideration in mUC to ensure that the opportunity to use targeted therapies is not missed. The European Association of Urology (EAU) recommends testing for *FGFR3* alterations at the time of mUC diagnosis to guide treatment planning, including eligibility for clinical trials [55]. Testing later in the disease course may limit treatment options, as patients may experience deterioration in performance status or become ineligible for further treatment, not only due to disease progression but also to the turnaround time to obtain molecular results to select the next treatment line. An alternative strategy could be to perform *FGFR3* testing while patients are undergoing immunotherapy-based treatment, as this may enable a faster transition to FGFR-targeted treatment upon disease progression.

Incorporating FGFR testing into routine diagnostic workflows for mUC, along with PD-L1 expression testing and other biomarkers (such as HER2) ensures that results are available in a timely manner for making informed treatment decisions. But implementing this recommendation may not be straightforward and will ultimately depend on each institution’s capacity and resource availability. Institutions with limited resources may prioritize testing patients currently receiving immunotherapy. Although biomarker testing is essential for guiding the appropriate selection of systemic, targeted therapies and for informing subsequent treatment strategies, patients with mUC are often under-tested. This is largely due to non-standardized testing practices and prolonged turnaround times associated with traditional, oncologist-initiated testing workflows. In this context, reflex testing- initiated automatically by the pathology department at the time of diagnosis - should be considered the optimal approach to ensure timely and consistent biomarker assessment, thereby facilitating more effective treatment planning.

When considering the timing of testing, obtaining tissue at the time of progression may be challenging, either due to technical difficulties in performing the biopsy or the patient’s clinical status. It should be emphasized that the sample obtained at diagnosis is appropriate for testing. If a tissue sample is not available or is insufficient, liquid biopsy could be an alternative, although its use in this setting is still exploratory (see Section 9).

## 7. How to Test for FGFR Alterations

Several methods are available to identify *FGFR* alterations, each with advantages and limitations depending on the clinical context, type of alteration being investigated, and resources of the healthcare setting.

Due to the diversity of copy number alterations (CNAs), single nucleotide variants (SNVs), and gene fusions in the mUC genome, testing methods that target only one type of alteration (e.g., PCR or FISH) may be less suitable. Due to its ability to identify a wide range of alterations—including CNAs, SNVs, and gene fusions—and provide a comprehensive, sensitive, and actionable tumor molecular profile, NGS is the preferred method for testing *FGFR* alterations [46,48]. In addition to its high sensitivity and specificity, NGS enables multiplex testing, allowing simultaneous evaluation of multiple genes and alterations in a single assay, and is able to detect genomic alterations in formalin-fixed, paraffin-embedded tumor tissue and circulating tumor DNA (ctDNA) [48,56]. While the upfront cost of NGS may be higher than other techniques, its ability to test for multiple alterations in one assay, sparing time and costs, makes it ultimately more cost-effective.

Various multigene panels are currently available for targeted sequencing, covering a spectrum from a few to several hundred genes (Table 2). These panels may employ DNA and/or RNA sequencing for the detection of gene fusions. Because intronic regions can hinder the reliable identification of fusions when using DNA-based methods, RNA sequencing generally provides superior sensitivity and coverage for gene fusion detection [17]. The ESMO has recently updated its recommendations on the use of tumor NGS in routine clinical practice for patients with advanced cancers. These guidelines support the use of NGS, not only for tumor type-specific genomic profiling, but also for the detection of tumor-agnostic alterations with available matched therapies, among which *FGFR* alterations are included [45].

**Table 2:** Overview of NGS assays.

Name	Input	Advantages	Limitations
Amplicon-based assay	10–20 ng of DNA and/or RNA	Suitable for sensitive analysis of specific regions of interest, such as known mutation hotspots or selected genes.	Limitations in fusion analysis and CNV detection. Limited ability to detect gene fusions when the fusion partner is unknown or when intronic regions are large and complex.
Hybrid capture-based assay	~50 ng of DNA and/or RNA	Suitable for detecting hotspot mutations and entire coding regions of genes. Enables broader fusion analysis, including rare partners. Improved CNV detection. When combined with UMIs, can reliably detect variants at an allele frequency below 5%. Allows detection of gene fusions even when the fusion partner is unknown.	Higher nucleic acid input requirements. Lower sensitivity for detecting low-frequency variants.

CNV, copy number variation; DNA, deoxyribonucleic acid; ng, nanogram; RNA, ribonucleic acid; UMI, unique molecular identifier.

RT-PCR is another potential method for detecting *FGFR* alterations. The Therascreen^®^ FGFR RGQ RT-PCR Kit (QIAGEN GmbH, QIAGEN Strasse 1, 40724 Hilden, Germany.) has been approved by the FDA and the European Medicines Agency (EMA) as a companion diagnostic test to identify mUC patients with *FGFR3* mutations and fusions in tumor tissue who are eligible for treatment with erdafitinib [57]. This method provides a rapid and reliable way to detect molecular alterations with a faster turnaround time than broader technologies, such as NGS, and is less expensive and simpler than comprehensive sequencing methods (Table 3). However, RT-PCR is limited to detecting predefined *FGFR* mutations and fusions, despite being sensitive and specific for its intended molecular targets. Unique primers are required to identify each fusion partner; therefore, the fusion partner and the location of the fusion breakpoint must be known prior to testing.

**Table 3:** Comparison of RT-PCR and NGS methods.

	RT-PCR	NGS
** Turnaround time **	3–5 days	5–21 days
** Type of *FGFR* alteration detected **	Pre-identified mutations and fusions	Any existing mutation with DNA sequencing Fusions with DNA or RNA sequencing (depending on the assay).
** Cost **	Low overall cost	Higher overall cost

DNA, deoxyribonucleic acid; FGFR, fibroblast growth factor receptor; RNA, ribonucleic acid. Erdafitinib is approved for variants that can be detected by the Therascreen^®^ assay, as used in the phase 3 THOR trial.

IHC is a widely accessible and cost-effective method. However, it is not currently recommended for the detection of *FGFR* alterations and relies on the assessment of FGFR protein overexpression. Although it requires less tissue compared with more comprehensive techniques such as NGS—an advantage in cases with limited material—, the lack of concordance between local and central results and the lack of standard cutoffs for *FGFR3* assessment [29], with heterogeneous methods used by different authors [32,58], suggest that this method still requires optimization. Standardization of IHC protocols and FGFR3 expression scoring across laboratories is essential to ensure reproducibility and accuracy of results. Therefore, while *FGFR3* mutation status can inform clinical decision-making, protein expression currently lacks predictive and prognostic value [37]. Importantly, FGFR3 overexpression is not associated with prognosis in *FGFR3* wild-type tumors, suggesting that *FGFR3* mutations exert distinct functional effects compared with protein overexpression [37]. Additional evidence is required to establish whether IHC could have a role in selecting patients with mUC for FGFR-targeted therapies.

FISH has limitations when used to identify *FGFR* alterations. Despite its good sensitivity and specificity in detecting *FGFR3* fusions, FISH has a limited detection range and is not suitable for detecting point mutations. This results in incomplete *FGFR3* characterization and lower resolution compared to sequencing methods. Technical challenges, such as the requirement for adequately preserved tissue samples (as with NGS) and subjectivity in result interpretation, which can contribute to false-positive and false-negative findings, limit the reliability of FISH as a method for detecting *FGFR* alterations in mUC [44,59].

Finally, Sanger sequencing can be used to identify point mutations and, in settings where neither NGS nor RT-PCR are available, may be considered to identify *FGFR* alterations in combination with FISH for detection of gene fusions. However, its clinical applicability in mUC is limited due to its low sensitivity for low-frequency variants (VAF < 15–20%), restricted multiplexing capacity, and the need for relatively large amounts of high-quality DNA [60,61].

Overall, comprehensive genomic profiling using NGS is the preferred method for accurately identifying actionable *FGFR* alterations. Testing for *FGFR3* mutations and fusions can be performed at DNA and RNA levels, with the latter presumably providing higher sensitivity. However, the choice of method may also depend on tissue quality, with RNA-based tests being particularly sensitive to degradation in poorly preserved samples [58]. Of note, the nucleated tumor cell content in the sample is of paramount importance and may vary depending on the assay used. If the percentage of nucleated tumor cells is lower than what the assay requires, microdissection should be performed to not compromise the assay’s sensitivity.

## 8. How to Report FGFR Alterations

The reporting of FGFR test results should comply with best practices in molecular diagnostics to ensure accurate interpretation and to optimally guide therapeutic decisions and patient management.

The report should provide a clear and comprehensive summary of the histopathologic and molecular findings, together with their clinical implications. When histopathologic and molecular analyses are performed in different laboratories, correct sample identification and morphological verification are essential to ensure accurate macrodissection of tumor cell-enriched areas.

The description of the analyzed sample should specify its type (e.g., tumor tissue biopsy, cytology specimen, surgical resection, or blood, plasma, or urine sample). For tissue samples, the anatomic site of origin, whether the sample is from a primary or metastatic tumor, and the preservation method (e.g., formalin-fixed paraffin-embedded [FFPE] or frozen) should be indicated. A pathology-based assessment of tumor cell content and, when feasible, histologic subtype should also be included, as this information is essential to avoid misinterpretation related to second primary tumors or other confounders [47].

The report should include a concise summary of the methodology used. Specifically, it should clearly state the type of test performed (e.g., NGS, RT-PCR, or other) and describe the technical approach, including whether the test targeted specific FGFR genes (e.g., *FGFR3*) and whether it evaluated mutations, amplifications, or fusions. While not mandatory, the report may also include relevant platform-specific details, such as coverage of specific exons or genes, if applicable.

Because most laboratories performing molecular assays for clinical use have limited access to the patient’s medical history, genomic reports are often generated without critical clinical information, such as prior treatments, which may influence the interpretation of genomic biomarkers and their clinical significance. To address this limitation, the report should indicate the reason for testing and include any additional clinical context provided to the laboratory.

The results section should clearly describe the *FGFR* alteration(s) identified, including with the type of alteration (e.g., mutation, fusion, or amplification), whenever possible using the Human Genome Variation Society guidelines. For mutations, details such as the specific nucleotide substitution and corresponding amino acid change should be reported, together with the gene transcript used for the variant description. For amplifications, the amplification status and, if available, the estimated fold-change should be provided. For fusions, both the FGFR gene involved and its fusion partner should be specified. When reporting point mutations, the variant allele frequency (VAF) should be included as it provides insight into the proportion of tumor cells harboring the alteration. Each alteration should be classified according to its known or potential clinical significance (e.g., pathogenic, likely pathogenic, or variant of uncertain significance [VUS]). Additionally, the report may include alterations that were tested for but not detected, especially if they are clinically relevant (e.g., “No *FGFR3* fusions detected”).

The interpretation section should provide a clinically oriented summary of the molecular findings. This includes whether the *FGFR* alteration is actionable, its relevance to FGFR-targeted therapies (e.g., eligibility for erdafitinib), and any known resistance mechanisms or therapeutic implications of the specific alteration. If supported by published evidence, the report should also state whether the detected *FGFR* alteration has prognostic significance.

A critical component of the report is the section addressing the therapeutic implications of the identified alterations. This section should summarize how the molecular findings may influence patient management by confirming or excluding eligibility for FGFR-targeted therapies, discussing whether the alteration is associated with resistance to standard treatments, and indicating whether the patient may be an appropriate candidate for clinical trials investigating FGFR-targeted agents.

The final section of the report should propose follow-up actions and actionable measures. This may include additional testing, if clinically relevant (e.g., reflex RNA sequencing to clarify fusion status), referral to a molecular tumor board for expert interpretation of complex or uncertain findings, and explicit treatment recommendations, such as initiation of FGFR-targeted therapy or referral for enrollment in a clinical trial.

A “Notes” section may also be included to provide information on assay performance metrics (e.g., specific alterations not covered, sensitivity thresholds, or sample quality issues) and, for tissue samples, sample quality, encompassing the percentage of nucleated tumor cells (including the use of tumor cell enrichment techniques such as macro- or microdissection), DNA quality, and the extent of necrosis. When the neoplastic cell content falls below the required threshold, the report should specify whether biomarker-negative results should be considered inconclusive. In cases where no alterations are detected, this information helps distinguish negative results from non-diagnostic reports and guides the next steps, such as the need for additional testing.

Structuring the FGFR test report to systematically incorporate these components ensures that clinicians receive all the information necessary to interpret the results appropriately, make informed therapeutic decisions, and optimize patient care.

### General Summary of Key Testing Recommendations

- *FGFR3* testing should ideally be performed at diagnosis, using a reflex testing approach.
- If *FGFR3* testing is not feasible at diagnosis, it should be considered at disease progression following first-line treatment.
- FFPE tissue is the preferred specimen type for *FGFR3* testing.
- Optimal formalin fixation is essential and should be performed for 6–48 h using neutral buffered formalin (pH 7), as non-buffered solutions may lead to tissue degradation. Overfixation should be avoided, and areas of necrosis should be carefully assessed.
- In samples with low tumor cellularity, tumor cell enrichment techniques, such as macrodissection, are recommended to preserve assay sensitivity.
- If tissue samples are unavailable or insufficient, liquid biopsy may be considered as an alternative; however, its use in this context remains exploratory.
- Comprehensive genomic profiling using NGS—including both DNA and RNA analysis—is the preferred method for accurately identifying actionable FGFR alterations, including mutations and gene fusions.
- RT-PCR represents an additional option for detecting FGFR alterations, including mutations and gene fusions (e.g., Therascreen^®^ FGFR RGQ RT-PCR Kit).
- Reports should be clear and concise and include confirmation of adequate tumor content; identification and clinical relevance of detected mutations or fusions (predictive of FGFR inhibitor sensitivity); VAF or fusion partner, when applicable; and a statement outlining assay limitations (e.g., fusion coverage).

## 9. Future Directions

The future of FGFR testing in mUC lies in the refinement and integration of advanced technologies that provide high sensitivity, accuracy, and ideally non-invasive approaches.

Liquid biopsy shows promise as a dynamic tool for real-time genomic profiling and may be an alternative to tissue biopsy in this setting in the future [40]. Data on the use of liquid biopsies to detect *FGFR* alterations in mUC are beginning to emerge but are still inconsistent. While some studies report similar detection rates of FGFR alterations with tissue- and plasma-based testing (approximately 19%) [61], others indicate a low accuracy of available circulating tumor DNA (ctDNA) assays, particularly in detecting gene fusions [41,62]. Another issue associated with the use of ctDNA in this setting is the low sensitivity of ctDNA assays in detecting low-frequency mutations, particularly in patients with low tumor burden. Methods such as droplet digital PCR (ddPCR) have been employed but may not consistently identify low-frequency mutations, which can lead to false-negative results [63]. Additionally, there is a lack of standardization in ctDNA collection, processing, and analysis across laboratories and studies. Overall, the use of ctDNA in clinical practice is currently limited.

Urine-based testing is another promising complementary tool for FGFR testing in UC, which may overcome the challenges of identifying *FGFR* alterations in tissue samples. Urinary tumor DNA (utDNA), which refers to tumor-specific DNA fragments found in urine, represents a promising source of tumor DNA in bladder cancer and can be obtained completely noninvasively [64]. Despite their potential, the clinical implementation of liquid biopsy-based approaches using ctDNA from plasma or urinary tumor DNA is currently limited by substantial methodological heterogeneity, underscoring the need for standardization across the entire testing workflow. A critical first step toward harmonization is the standardization of pre-analytical variables, which are a major source of variability. For plasma-based assays, this includes uniform protocols for blood collection (e.g., type of collection tubes), time to processing, centrifugation steps, storage temperature, and maximum allowable delays before DNA extraction. Similarly, for urine-based testing, harmonized procedures are needed regarding urine volume, timing of collection (e.g., first-morning versus random samples), use of preservatives, storage conditions, and processing timelines, given the rapid degradation of cell-free DNA in urine. At the analytical level, consensus on assay selection and performance requirements is essential. This includes defining minimum sensitivity thresholds for variant allele frequency detection, standardized reporting of assay limits of detection, and clear criteria for calling FGFR mutations and fusions, which remain particularly challenging in liquid-based assays. Equally important is the harmonization of bioinformatic pipelines and reporting standards, including uniform variant annotation, classification of clinical relevance, and transparent reporting of inconclusive or low-confidence results. Alignment with existing international recommendations for molecular reporting in solid tumors would facilitate clinical interpretation and comparability across studies. Finally, prospective validation within standardized clinical workflows is required. This includes head-to-head comparisons of tissue-, plasma-, and urine-based FGFR testing using harmonized protocols, as well as integration of liquid biopsy into longitudinal sampling strategies to assess concordance, temporal stability, and clinical utility. In the era of precision medicine, combining ctDNA and utDNA holds potential for guiding the optimal selection of patients for bladder-conserving approaches [65]. However, robust prospective data are still needed before these tools can be routinely incorporated into clinical practice [42].

Regarding the potential role of FGFR targeting earlier in the disease course (e.g., in the neoadjuvant/peri-operative setting of MIBC), while there is a biologically plausible rationale to explore this concept, it remains hypothesis-generating at present. From a biological standpoint, there is a theoretical rationale to explore FGFR inhibition earlier in the disease course [53,66]. On the one hand, *FGFR3* alterations are early oncogenic events, particularly in urothelial carcinogenesis, and are already present in a substantial proportion of non-metastatic disease, including NMIBC and a subset of MIBC [29,36,66]. On the other hand, tumors harboring *FGFR3* alterations tend to show a luminal molecular phenotype, often associated with lower immune infiltration and distinct biology compared with basal/squamous tumors [36,67,68,69]. These data suggest that FGFR-altered tumors may represent a biologically defined subgroup in which targeted therapy could be more effective earlier [36,53]. However, despite this rationale, it has no supporting evidence at present, since all high-level clinical evidence supporting FGFR inhibitors (e.g., erdafitinib) comes from the advanced/metastatic setting, particularly post-platinum and post-immunotherapy disease, as exemplified by the THOR trial [7,70]. Key limitations preventing immediate translation to the neoadjuvant setting include (i) the absence of randomized neoadjuvant trials demonstrating an improvement in pathologic complete response (pCR), downstaging benefits, or an event-free or overall survival advantage; and (ii) lack of comparative data with cisplatin-based neoadjuvant chemotherapy, which remains the current standard of care for eligible MIBC patients [71]. Overall, the primary aim of FGFR testing remains consistent across disease settings, as outlined in this review. As the manuscript focuses on testing strategies, these approaches can be readily adapted to neoadjuvant or preoperative settings, given that the sample types and analytical methodologies would largely remain unchanged. This adaptability ensures that irrespective of treatment timing, FGFR testing continues to support accurate patient selection for FGFR-targeted therapies.

## 10. Conclusions

*FGFR3* alterations are found in 15–20% of mUC, and the availability of FGFR inhibitors, such as erdafitinib, has made *FGFR3* testing a critical component of precision oncology for this tumor. Early testing (at the time of mUC diagnosis) is essential to avoid missing therapeutic opportunities due to clinical deterioration later in the disease course. NGS is the preferred testing method for the detection of clinically relevant *FGFR* alterations due to its ability to detect a wide range of genomic changes (namely, point mutations and fusions) in a single assay. FGFR test reports should be comprehensive and clearly structured, providing details on sample type and quality, testing methodology, identified alterations, clinical significance and interpretation, implications for targeted therapy, and clear follow-up recommendations. Implementing institutional workflows that enable routine molecular profiling in mUC, including access to NGS and timely reporting, should be a priority as it has the potential to improve patient outcomes.

## Data Availability

No new data were created or analyzed in this study.
